# Luminescence Properties of Eu^3+^, Ba^2+^, and Bi^3+^ Co-Doped YVO_4_ for Wide-Spectrum Excitation

**DOI:** 10.3390/nano15181444

**Published:** 2025-09-19

**Authors:** Jianhua Huang, Cong Dong, Ping Huang, Wei Zhong, Yinqi Luo, Jianmin Li, Yibiao Hu, Wenjie Duan, Lingjia Qiu, Wenzhen Qin, Yu Xie

**Affiliations:** 1 Jiangxi Provincial Key Laboratory of Power Batteries & Energy Storage Materials, Xinyu University, Xinyu 338000, China; 15559770955@163.com (C.D.); ph0603@163.com (P.H.); zhongweiwen@126.com (W.Z.); luoyq1993@gmail.com (Y.L.); ljm6729126@163.com (J.L.); 19070103006@163.com (Y.H.); 2 Hunan Engineering Laboratory for Control and Optimization of PV Systems, Hunan Vocational Institute of Technology, Xiangtan 411100, China; wenjiesky@foxmail.com; 3 College of Environment and Chemical Engineering, Nanchang Hangkong University, Nanchang 330063, China; lingjiaqiu0701@sina.com

**Keywords:** Eu^3+^, Ba^2+^, Bi^3+^ co-doped YVO_4_, sol–gel method, wide-spectrum excitation, luminescence properties

## Abstract

YVO_4_ based phosphors have aroused extensive interest in the field of optoelectronics due to their good chemical stability and unique luminescence properties. However, commercialization of YVO_4_ phosphors requires high luminescence intensity, enhanced conversion efficiency, and a wide excitation spectrum. In this work, Eu^3+^, Ba^2+^, Bi^3+^ co-doped YVO_4_ was prepared by the sol–gel method. The XRD of YVO_4_: 5%Eu^3+^, 5%Ba^2+^, 0.5%Bi^3+^ phosphor analysis confirms the pure tetragonal phase, with a fairly large size of approximately 100 nm for the optimal composition. And the SEM and TEM revealed well-dispersed spherical nanoparticles with sizes of 100–120 nm. The introduction of Ba^2+^ ions enhanced the luminescence intensity, while the incorporation of Bi^3+^ ions improved the excitation width of the phosphor. The resulting YVO_4_: 5%Eu^3+^, 5%Ba^2+^, 0.5%Bi^3+^ phosphor exhibited a 1.39-times broader excitation bandwidth and a 2.72-times greater luminescence intensity at 618 nm compared to the benchmark YVO_4_: 5% Eu^3+^ sample. Additionally, the transmittance of the films in the 350 nm to 800 nm region exceeded 85%. The YVO_4_: 5%Eu^3+^, 5%Ba^2+^, 0.5%Bi^3+^ film effectively absorbed ultraviolet light and converted it to red emission, enabling potential applications in solar cell window layers, dye-sensitized cell luminescence layers, and solar cell packaging glass.

## 1. Introduction

YVO_4_ phosphor with a tetragonal phase shows excellent chemical stability, such as a high decomposition temperature (>1800 °C) and high resistance to moisture and common solvents, and unique luminescence properties. In particular, the Eu^3+^-doped YVO_4_ red emission phosphor has received extensive interest for many applications [1,2,3,4,5,6,7,8]. Compared with traditional Y_2_O_3_: Eu^3+^ phosphors, YVO_4_: Eu^3+^ phosphor exhibits significantly higher luminescence intensity (reportedly about 2–3 times higher under UV excitation), superior color purity, and a much wider operating temperature range, maintaining high efficiency up to temperatures exceeding 200 °C [1,2,3,4].

Zuo Y Y et al. [9,10,11,12,13,14,15] found that Bi^3+^-doped YVO_4_: Eu^3+^ can broaden the band edges of the absorption and excitation spectrum and red-shift the absorption edge and excitation edge. However, uneven doping and concentration quenching of Bi^3+^ led to a reduction in photoluminescence intensity. Wangkhem R et al. [16,17,18,19,20] found that the doping of M^2+^ alkaline ions into YVO_4_: Eu^3+^ introduced radiation defect centers and enhanced the luminescence intensity. L. Jiang et al. [21] improved the luminescence intensity by coating YVO_4_: Eu^3+^ with SiO_2_. Enrico C et al. [22] prepared YVO_4_: Ln^3+^ (Ln^3+^ = Eu^3+^, Sm^3+^, and Dy^3+^) phosphors, and studied the effect of co-doped ions on their luminescence properties. Tang L et al. [23] prepared YVO_4_: Eu^3+^-trifluoro acetone by inorganic and organic hybridization, which showed significantly enhanced luminescence intensity. Wang GF et al. [24] synthesized water-soluble YVO_4_: Ln^3+^ and YVO_4_: Ln^3+^, Ba^2+^ (Ln = Ce, Dy, Eu, Sm) nanocrystals by the polyethylpyrrolidone-assisted hydrothermal method, and found that Ba^2+^ doping enhanced the luminescence intensity. Park K C et al. [25] found that in Ba_3(1-x)_Eu_2x_V_2_O_8_, the luminous intensity of VO_4_^3−^ decreased with the increase in Eu^3+^ content due to the charge transfer transition of VO_4_^3−^. The excitation spectra of Ba_2.1_Eu_0.6_V_2_O_8_ only showed charge transfer transition and f-f transition of Eu^3+^ ions. In the Ba_3(1−x)_Y_2x_V_2_O_8_ (0 ≤ x ≤ 0.33) system, the luminescence intensity decreases with the increase in Y^3+^ content. YVO_4_: Eu^3+^ phosphors synthesized by the high-temperature solid phase method usually exhibit severe grain agglomeration. The crystal destruction and incomplete shape obtained upon ball milling result in decreased luminescence intensity and increased light decay. Moreover, uneven particles of YVO_4_: Eu^3+^ luminescent materials also result in uneven coating, and lead to a series of problems such as poor color temperature consistency, light color consistency, and color rendering index of the display. Traditional synthesis methods also cause low luminous intensity and light conversion efficiency of luminous materials. Therefore, single rare earth and alkaline earth-doped YVO_4_: Eu^3+^ luminous materials have narrow excitation spectra, which hinders their commercial applications [26]. Several recent studies have reported on novel hybrid luminescent materials based on YVO_4_ hollow mesoporous microspheres [27], dynamic color gradient anti-counterfeiting technology with multi-wavelength excitation [28], and nanoporous YVO_4_ as a luminescent system for molecular encapsulation detection [29]. These findings present broad application prospects of YVO_4_-based phosphors in the fields of optoelectronic integration, anti-counterfeiting technology, and optical sensing. Developing improved YVO_4_: Eu^3+^ phosphors can significantly improve luminescence efficiency and anti-counterfeiting capabilities and also open up new research pathways for developing novel rare-earth complexes.

Although single-doped or double-doped Ba^2+^ or Bi^3+^ have been reported, how to simultaneously solve the two key problems of a “narrow excitation spectrum” and “luminescence intensity needs to be improved” faced by YVO_4_: Eu^3+^ materials through the synergistic effect of ternary co-doping (Synergistic Effect) has yet to be deeply explored. In this work, YVO_4_: Eu^3+^, Ba^2+^, Bi^3+^ crystals and films were synthesized by the sol–gel method. In this material, Eu^3+^ ion serves as the activator, dominating the red emission of phosphor. Ba^2+^ ion enhances luminescence, while the introduction of Bi^3+^ ion broadens the excitation spectrum of the samples. The effects of annealing temperature, doping concentration, and coating parameters on the phase structure, microstructural characteristics, and photoluminescence properties of crystals and films were systematically investigated. Additionally, the interaction mechanisms of Eu^3+^-, Ba^2+^-, and Bi^3+^-doping ions were explored, including their roles in morphology control, wide-spectrum excitation, and luminescence enhancement.

## 2. Experimental

### 2.1. Materials

Yttrium nitrate (Y(NO_3_)_3_·6H_2_O, 99.99%), europium nitrate (Eu(NO_3_)_3_·6H_2_O, 99.99%), barium nitrate (Ba(NO_3_)_2_, 99.99%), bismuth nitrate (Bi(NO_3_)_3_, 99.99%), ammonium vanadate (NH_4_VO_3_, 99.99%), and citric acid (C_6_H_8_O_7_) (analytical grade, A.R.) were used as the raw materials. Dilute nitric acid, acetone (CH_3_COCH_3_) (analytical-grade, A.R.), ammonia water (NH_3_·H_2_O) (analytical-grade, A.R.), polyethylene glycol (PEG 4000) (analytical grade, A.R.), and ethanol (C_2_H_5_OH) (analytical-grade, A.R.) were used as the solvents. All chemicals were purchased from Shanghai Macklin Biochemical Co., Ltd. (Shanghai, China). All chemicals were used as received, without further purification. Deionized water was used throughout this work.

### 2.2. Synthesis of YVO_4_: xEu^3+^, yBa^2+^, and zBi^3+^ Crystals

The preparation process of YVO_4_: 1%Eu^3+^ crystal is as follows. First, 4.95 mmol of Y(NO_3_)_3_·6H_2_O and 0.05 mmol of Eu(NO_3_)_3_·6H_2_O were dissolved in H_2_O (10 mL), and the mixture was stirred evenly. Then, citric acid (metal ion–citric acid = 1:3) was added at a constant temperature of 80 °C with stirring to form solution A. NH_4_VO_3_ (5.0 mmol) and NH_3_·H_2_O (0.5 mL) were added into 20 mL of deionized water. After heating and stirring at a constant temperature of 90 °C, a transparent yellow solution was formed, which was named solution B. Solution B was slowly added into solution A to form mixed solution C. At this time, the color of mixed solution C changed from brick red to dark red (A and B were mixed after cooling) or dark green (A and B were mixed before cooling). After complete addition of solution B, the mixed solution C was ultrasonicated for 10 min. The pH value of the mixed solution C was accurately adjusted to 7.0 using concentrated ammonia (25–28%) (measured using a pH meter), and a blue, uniform, and transparent solution D was obtained. Solution D was continuously stirred at a constant temperature of 80 °C to slowly form a sol. As the reaction time continued, it gradually formed a thick, blue gel. The obtained gel was dried at 120 °C for 6 h. After drying, a honeycomb-shaped dark blue dry gel was obtained. The dry gel was calcined in a muffle furnace at 500 °C for 3 h, and the organic matter in the dry gel was removed to obtain a yellowish YVO_4_: 1%Eu^3+^ powder sample. The calcined YVO_4_: 1%Eu^3+^ samples were annealed for 2 h at 800 °C, 900 °C, 1000 °C, 1100 °C, and 1200 °C, respectively, to obtain white powder samples.

According to the preparation scheme of YVO_4_: 1%Eu^3+^ samples, a series of YVO_4_: xEu^3+^ (x = 3%, 5%, 7%), YVO_4_: 5%Eu^3+^, yBa^2+^ (y = 1%, 3%, 5%, 7%) and YVO_4_: 5%Eu^3+^, 5%Ba^2+^, zBi^3+^ (z = 0.5%, 1%, 1.5%, 2%) samples were prepared by adjusting the concentration of solute in solution A according to the doping ratio. The samples were finally annealed at 1100 °C for 2 h.

### 2.3. Synthesis of YVO_4_: Eu^3+^, Ba^2+^, and Bi^3+^ Films

The preparation process of YVO_4_: Eu^3+^, Ba^2+^, and Bi^3+^ films was similar to that of YVO_4_: 5%Eu^3+^, 5%Ba^2+^, and 0.5%Bi^3+^ crystals. After obtaining the uniform blue solution D, 0.06 g/mL of polyethylene glycol (PEG 4000) was added to the blue solution. After stirring for several hours under heated conditions, the gel was obtained. Quartz glass was chosen as the substrate for the film. The substrate was soaked in HNO_3_ in advance. The mixed solution was prepared according to the ratio of C_2_H_5_OH:H_2_O:CH_3_COCH_3_ = 1:1:1. The quartz substrate was soaked in the mixed solution for 30 min and dried before use. The film was coated on the quartz substrate by the rotating coating method (rotating at 4000 rpm for 30 s with a spinner). The gel was dropped on the quartz substrate using a 1 mL dropper, and then the film was formed by rotating the coating at a rotation speed of 4000 r/min for 30 s. After each layer of coating rotation, the wet film was placed into the oven at 80 °C for 10 min. After the film was formed, rotating coating was continued and the steps were repeated to prepare the film with 1, 3, 5, 7, and 9 layers. Finally, the prepared film was calcined in a muffle furnace, and the film sample was obtained by pre-sintering at 500 °C for 3 h and then at 1100 °C for 2 h.

### 2.4. Characterization

Unless otherwise specified, all measurements were repeated on three independently synthesized batches of samples to ensure reproducibility. The data presented are the average values. X-ray diffraction (XRD, Bruker D8 Advance, Karlsruhe, Germany), scanning electron microscopy (SEM, S-4800, Tokyo, Japan), and transmission electron microscopy (TEM, Tecnai G2 F20 S-TWIN, USA) were used to investigate the phase structures and microstructural characteristics of YVO_4_: Eu^3+^, Ba^2+^, and Bi^3+^ crystals and films. ICP-OES: Thermo Fisher iCAP 7400 (Thermo Fisher Scientific, Waltham, MA, USA). Photoluminescence excitation and emission spectra were recorded with a Hitachi F-4600 fluorescence spectrophotometer (Hitachi, Tokyo, Japan) equipped with a xenon lamp source. UV–Vis absorption spectra were monitored with a UV3150 spectrophotometer (Shimadzu, Japan). All measurements were conducted in air at room temperature.

## 3. Results and Discussion

### 3.1. Photoluminescence Properties

**Figure 1:**
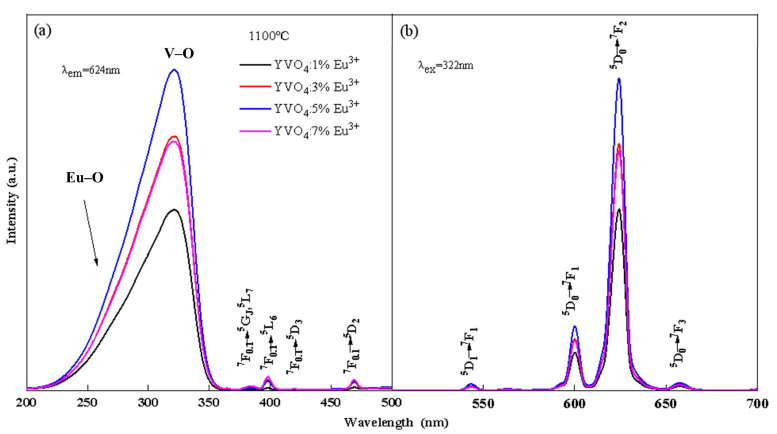
Excitation (**a**) and emission (**b**) spectra of YVO_4_: xEu^3+^ (x = 1%, 3%, 5%, 7%) after annealing at 1100 °C.

**Figure 2:**
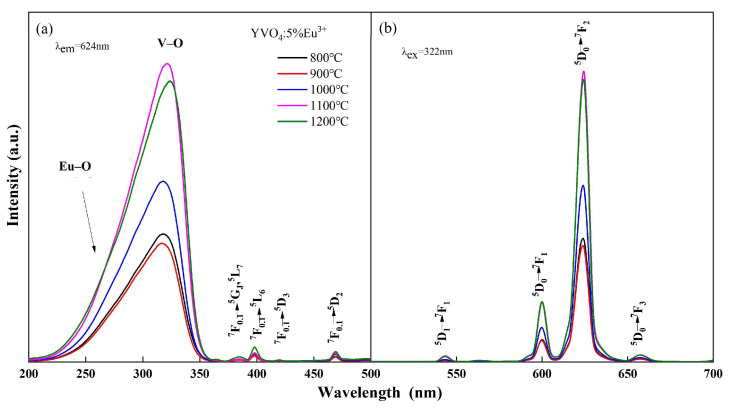
Excitation (**a**) and emission (**b**) spectra of YVO_4_: 5%Eu^3+^ annealed at different temperatures.

The photoluminescence spectra of YVO_4_: Eu^3+^ crystals with different doping concentrations annealed at 1100 °C and the crystals with 5% Eu^3+^ concentration annealed at different temperatures are shown in Figure 1 and Figure 2. The peak at 260 nm (Figure 1a and Figure 2a) is generated by the Eu–O charge transfer band, and the peak at 320 nm is generated by the charge transfer from the oxygen ligand to the vanadium ion center [30,31]. The ^7^F_0.1_ → ^5^G_J_, ^5^L_7_ transition at 384 nm, ^7^F_0.1_ → ^5^L_6_ transition at 395 nm, ^7^F_0.1_ → ^5^D_3_ transition at 420 nm, and ^7^F_0.1_ → ^5^D_2_ transition at 470 nm are all characteristic excitation peaks of Eu^3+^. The ^5^D_1_ → ^7^F_1_ transition at 538 nm, ^5^D_0_ → ^7^F_1_ transition at 600 nm, ^5^D_0_ → ^7^F_2_ transition at 618 nm, and ^5^D_0_ → ^7^F_3_ transition at 657 nm [32,33] are all characteristic emission peaks of Eu^3+^ (Figure 1b and Figure 2b). The above results showed that Eu^3+^ was successfully doped into the YVO_4_ matrix by the sol–gel method.

The luminescence intensity of YVO_4_: Eu^3+^ crystals varied with the annealing temperature and doping concentration, as seen from Figure 1 and Figure 2. When the annealing temperature reached 1100 °C, the excitation intensity was 2.28 times that of 800 °C, and the emission intensity at 618 nm was 2.25 times that of 800 °C. When the doping concentration was 5%, the excitation intensity and emission intensity reached the maximum. In the band range of 200–350 nm, the excitation spectral intensity at 5% doping was 1.73 times that of 1%, 1.24 times that of 3%, and 1.26 times that of 7%. The luminescence intensity at 618 nm at 5% doping was 1.72 times that of 1%, 1.27 times that of 3%, and 1.31 times that of 7%.

The change in luminescence properties of YVO_4_: 5%Eu^3+^ caused by the variation in annealing temperature can be attributed to several factors. As the annealing temperature increases, the covalent bond interaction strengthens, which facilitates the electron migration from O^2−^ to V^5−^. This results in a slight red-shift in the V–O electron migration band [34] and enhanced luminescence intensity. Additionally, the improvement in crystallinity with the increase in temperature reduces crystal defects and non-radiative traps, leading to enhanced luminescence intensity [35]. Furthermore, the rapid growth of crystals at higher temperatures results in the formation of submicron- to micron-sized crystals with varying sizes and reduced uniformity, creating more pronounced inter-crystal voids. Consequently, the photoluminescence performance of these micron-sized luminescent materials was inferior to that of nanomaterials.

When the doping concentration of Eu^3+^ reached 5% and then continued to increase, the luminescence intensity of YVO_4_: Eu^3+^ crystals decreased. The reason is that the spacing between the particles gradually decreased as the Eu^3+^ concentration continued to increase to a certain critical point. When the spacing was less than 1–2 nm, the concentration quenching phenomenon occurred, resulting in a reduction in luminescence intensity [36].

**Figure 3:**
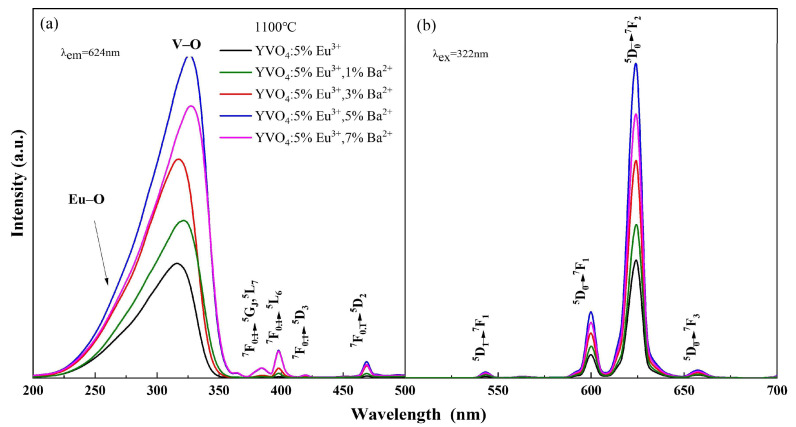
Excitation (**a**) and emission (**b**) spectra of YVO_4_: 5%Eu^3+^, yBa^2+^ (y = 1%, 3%, 5%, 7%).

The photoluminescence intensity of YVO_4_: 5%Eu^3+^, yBa^2+^ crystals varied with the doping concentration, as seen in Figure 3a,b. When the concentration of Ba^2+^ was 5%, the excitation intensity of YVO_4_: 5%Eu^3+^, 5%Ba^2+^ crystals at 200–350 nm was 2.89 times that of undoped Ba^2+^, and the luminescence intensity of ^5^D_0_ → ^7^F_2_ at 618 nm was 2.72 times that of undoped Ba^2+^. As the concentration of Ba^2+^ continued to increase, the excitation intensity and luminescence intensity of YVO_4_: Eu^3+^, Ba^2+^ crystals decreased. This decrease occurred because the radius of Ba^2+^ is larger than that of Eu^3+^, and there is an energy transfer between Ba^2+^ and Eu^3+^. In the non-radiative transition process, Ba^2+^ absorbs more energy than Eu^3+^. When there is competition between VO_4_^3−^-Eu^3+^ energy transfer and Eu^3+^-Ba^2+^ non-radiative transition, the increase in Ba^2+^ doping concentration leads to concentration quenching and a decrease in luminescence intensity [37]. The above results confirmed that Ba^2+^ was successfully doped into the YVO_4_: Eu^3+^ matrix by the sol–gel method.

Ba^2+^ doping promoted a slight red-shift in the V–O electron migration band of the excitation spectrum (Figure 3a). This is because the charge radius ratio of Ba^2+^ is smaller than that of Y^3+^, and the binding of Ba^2+^ with the oxygen atom is less tight than that of Y^3+^ with the oxygen atom. When Ba^2+^ was doped into the lattice, lattice parameters changed, resulting in a looser binding of Y^3+^/Ba^2+^ with the oxygen atom in the lattice. Consequently, Ba^2+^ doping caused a slight lattice distortion. Thus, the charge transfer state (CTS) absorption band [38] was enhanced and the luminescence intensity of the CTS excitation band increased [39].

**Figure 4:**
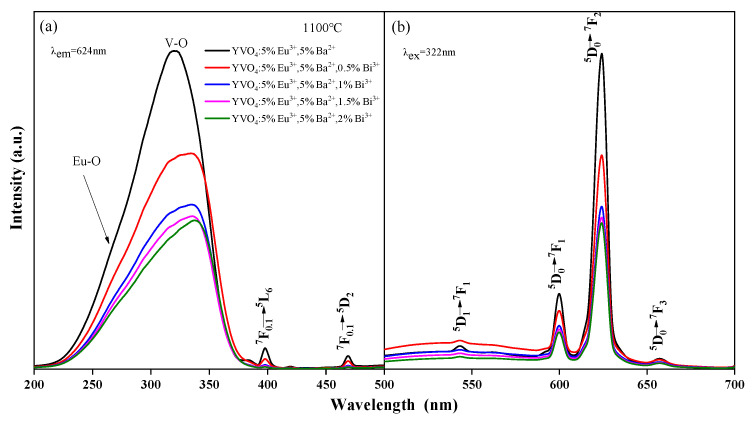
Excitation (**a**) and emission (**b**) spectra of YVO_4_: 5%Eu^3+^, 5%Ba^2+^, zBi^3+^ (z = 0.5%, 1%, 1.5%, 2%).

With the incorporation of Bi^3+^, the wide-spectrum excitation performance was considerably improved (Figure 4a). The excitation widths of YVO_4_: 5%Eu^3+^, 5%Ba^2+^, zBi^3+^ (z = 0.5%, 1%, 1.5%, 2%) crystals at 200–350 nm were 1.34 times, 1.29 times, 1.25 times, and 1.32 times that of YVO_4_: 5%Eu^3+^, 5%Ba^2+^, respectively. However, with the increase in Bi^3+^ doping concentration, the excitation intensity and luminescence intensity of YVO_4_: 5%Eu^3+^, 5%Ba^2+^, zBi^3+^ (z = 0.5%, 1%, 1.5%, 2%) crystals gradually decreased (Figure 4a,b) [40]. The excitation intensity of YVO_4_: 5%Eu^3+^, 5%Ba^2+^, 0.5%Bi^3+^ crystals at 200–350 nm was 0.73 times that of YVO_4_: 5%Eu^3+^, 5%Ba^2+^, and their luminescence intensity at 618 nm was 0.725 times that of YVO_4_: 5%Eu^3+^, 5%Ba^2+^. The excitation intensity of YVO_4_: 5%Eu^3+^, 5%Ba^2+^, 0.5%Bi^3+^ crystals at 200–350 nm was 2.11 times that of YVO_4_: 5%Eu^3+^, and the excitation width was 1.39 times that of YVO_4_: 5%Eu^3+^. Their luminescence intensity at 623 nm was 1.97 times that of YVO_4_: 5%Eu^3+^.

After the addition of Bi^3+^, a slight red-shift was observed in both the main peak and edge of the excitation band (Figure 4a). The main peak moved from 320 to 335 nm. This was due to the absorption of Bi^3+^ in the ultraviolet region of 200–400 nm, and the presence of an electron transfer between Bi^3+^-V^5+^ ions and energy transfer to Eu^3+^. Bi^3+^ plays a sensitization role here. The Eu–O and V–O absorption peaks of YVO_4_: Eu^3+^, Ba^2+^, Bi^3+^ crystals both decreased, mainly because the energy between O^2−^-Eu^3+^ and O^2−^-V^5+^ was transferred to the O^2−^-Bi^3+^ electron transition. Consequently, the absorption peaks of O^2−^-Eu^3+^ and O^2−^-V^5+^ decreased [41,42,43]. Another reason for the reduced excitation peak strength was the difficulty doping YVO_4_ at high concentrations. The incorporation of Bi^3+^ caused lattice distortion, reducing the actual doping amounts of Eu^3+^ and Ba^2+^, thus lowering the excitation strength. Furthermore, ICP-OES ion occupancy analysis enables the determination of occupancy characteristics for Eu^3+^, Ba^2+^, and Bi^3+^ ions within the YVO_4_ host. ICP-OES analysis revealed the following ion incorporation efficiencies: In YVO_4_: 5% Eu^3+^, the actual Eu^3+^ content was determined to be 4.82%; for YVO_4_: 5% Eu^3+^, 5% Ba^2+^, measured values showed 4.79% Eu^3+^ and 4.44% Ba^2+^; while in YVO_4_: 5% Eu^3+^, 5% Ba^2+^, 0.5% Bi^3+^, the incorporated concentrations were quantified as 4.35% Eu^3+^, 4.84% Ba^2+^, and 0.28% Bi^3+^. ICP-OES analysis shows that the actual doping amount of Bi^3+^ (0.28%) is much lower than its nominal doping concentration (0.5%), and its doping efficiency (56%) is also significantly lower than that of Eu^3+^ (87%) and Ba^2+^ (97%). This phenomenon is mainly attributed to the large ion radius mismatch between Bi^3+^ and Y^3+^.

The incorporation of both Ba^2+^ and Bi^3+^ ions into YVO_4_: Eu^3+^ phosphors significantly enhanced their luminescence properties. Ba^2+^ doping induced lattice expansion and local distortion, which reduced the crystal field symmetry around Eu^3+^ and strengthened its electric dipole transition (^5^D_0_ → ^7^F_2_) [10,39], thereby improving red emission intensity. Concurrently, Ba^2+^ doping introduced positive charge defects, while Bi^3+^ doping partially compensated for charge imbalance, suppressing the formation of oxygen vacancies and other non-radiative recombination centers to minimize luminescence quenching. Additionally, the strong ultraviolet absorption (300–350 nm) of Bi^3+^ enabled efficient excitation energy harvesting and subsequent non-radiative energy transfer to Eu^3+^, thereby boosting excitation width and luminescence efficiency. Thus, the luminescence behavior of YVO_4_: Eu^3+^ under Bi^3+^ doping is a result of the competition between effective sensitization at lower concentrations and concentration quenching effects at higher doping levels.

All emission peaks in the spectra (Figure 3b and Figure 4b) were characteristic peaks of Eu^3+^, with no emission peaks attributable to Ba^2+^ or Bi^3+^. This indicated that Ba^2+^ and Bi^3+^ themselves did not emit light, and that the energy transfer between Eu^3+^ and VO_4_^3−^ groups was efficient. The doping of Ba^2+^ only improved the luminescence intensity of YVO_4_: Eu^3+^ [44,45,46]. This enhancement occurred because doped Ba^2+^ replaced the position of Y^3+^ in the crystal lattice, creating a negative potential BaY′ and altering the lattice parameters, which increased the luminescence intensity [44]. Additionally, the sensitizer Bi^3+^ transferred energy to the active ion Eu^3+^ after the electron transfer between Bi^3+^-V^5+^ ions.

**Figure 5:**
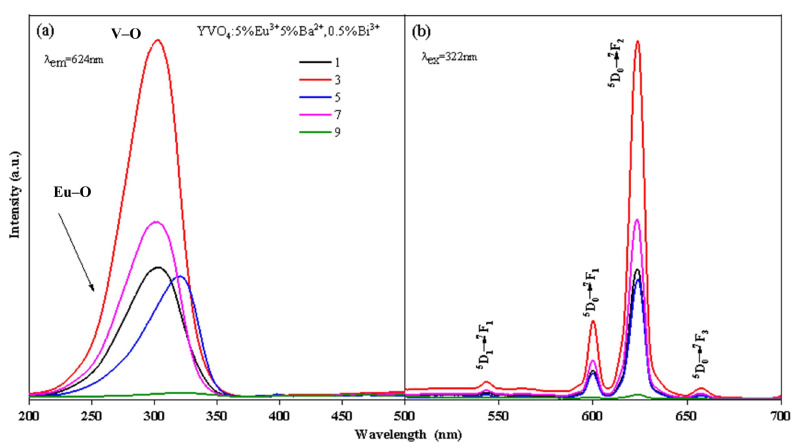
Excitation (**a**) and emission (**b**) spectra of YVO_4_: 5%Eu^3+^, 5%Ba^2+^, 0.5%Bi^3+^ films.

With the increase in the number of film layers, both the excitation intensity and luminescence intensity increased rapidly, reaching the maximum at the third layer (Figure 5a,b). However, as the number of film layers continued to increase, both the excitation intensity and luminescence intensity exhibited a decreasing trend. Specifically, the excitation strength of the film with 3 layers at 304 nm was 2.52, 2.94, 1.95, and 32.38 times that of the 1-layer, 5-layer, 7-layer and 9-layer films, respectively. Similarly, the luminescence intensity of 3-layer film at 618 nm was 2.62 times, 3.02 times, 2.00 times, and 48.52 times that of 1-layer, 5-layer, 7-layer, and 9-layer films, respectively.

### 3.2. Phase Structures and Microstructural Characteristics

**Figure 6:**
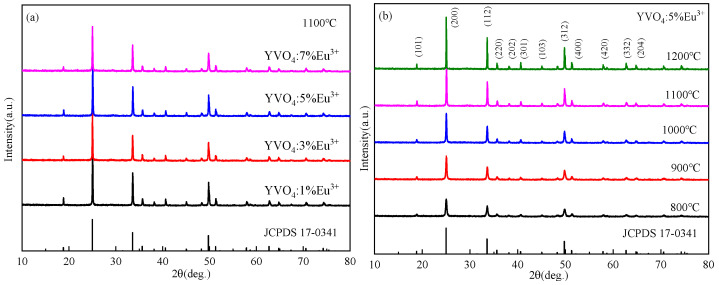
(**a**) XRD patterns of YVO_4_: 5%Eu^3+^ after annealing at different temperatures (JCPDS card No. 17-0341); (**b**) XRD patterns of YVO_4_: xEu^3+^ (x = 1%, 3%, 5%, 7%) after annealing at 1100 °C (JCPDS card No. 17-0341).

XRD patterns of YVO_4_: Eu^3+^ crystals prepared by the sol–gel method with different doping concentrations and the same doping concentration at different annealing temperatures are shown in Figure 6a,b. The positions and relative intensities of the diffraction peaks of the samples matched well with the standard card (JCPD 17-0341), and corresponded to the YVO_4_ crystal group. The crystals showed tetragonal zircon structure without any heterogenous phase. With the increase in annealing temperature, doping concentration, and number of film layers, the diffraction peaks became sharper, and the crystallinity of the samples improved. A synthesis temperature of 1100 °C and a heating rate of 20 °C/min were selected in this study through comprehensive optimization of crystallinity, doping efficiency, and process feasibility. The reaction temperature of 1100 °C ensures pure-phase crystallization of YVO_4_, eliminates interference from impurity phases on luminescent properties, and facilitates effective Eu^3+^ incorporation into Y^3+^ lattice sites to enhance doping efficiency. Simultaneously, this temperature enables complete decomposition of organic precursors, and reduces carbon residues and non-radiative recombination centers, thereby improving luminous efficiency. The heating rate of 20 °C/min accelerates nucleation kinetics, yielding uniform and fine crystallites (approximately 100–300 nm) with enhanced luminescence uniformity. The synergistic effects of an appropriate temperature and heating rate optimized crystallization and defect suppression, ultimately achieving excellent luminescent performance.

**Figure 7:**
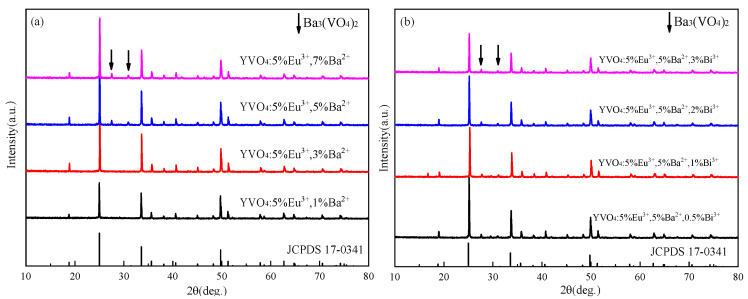
(**a**) XRD patterns of YVO_4_: 5%Eu^3+^, yBa^2+^ (y = 1%, 3%, 5%, 7%) after annealing at 1100 °C (JCPDS card No. 17-0341); (**b**) XRD patterns of YVO_4_: 5%Eu^3+^, 5%Ba^2+^, zBi^3+^ (z = 0.5%, 1%, 1.5%, 2%) after annealing at 1100 °C (JCPDS card No. 17-0341).

XRD patterns of YVO_4_: 5%Eu^3+^, yBa^2+^ and YVO_4_: 5%Eu^3+^, 5%Ba^2+^, zBi^3+^ at different Ba^2+^ doping concentrations are presented in Figure 7a,b, indicating the good crystallization properties of the samples. With the increase in Ba^2+^ doping concentration, Ba_3_(VO_4_)_2_ heterophase gradually appeared, and the diffraction peak shifted to a small angle (Figure 7a). This shift was due to the larger radius of Ba^2+^ (0.135 nm) compared to Y^3+^ (0.088 nm). At a low concentration, Ba^2+^ can effectively enter the lattice of YVO_4_ and replace the position of Y^3+^. However, at a high concentration, Ba^2+^ cannot mix well into the lattice of YVO_4_, which is consistent with some reports [47,48,49].

The diffraction peaks shifted towards smaller angles with the incorporation of Bi^3+^, and the heterodox peaks at 27.6° and 31.2° gradually increased in intensity with a higher Bi^3+^ doping concentration (Figure 7b). This indicates that at high doping concentrations, Bi^3+^ cannot be successfully incorporated into the lattice of YVO_4_.

**Figure 8:**
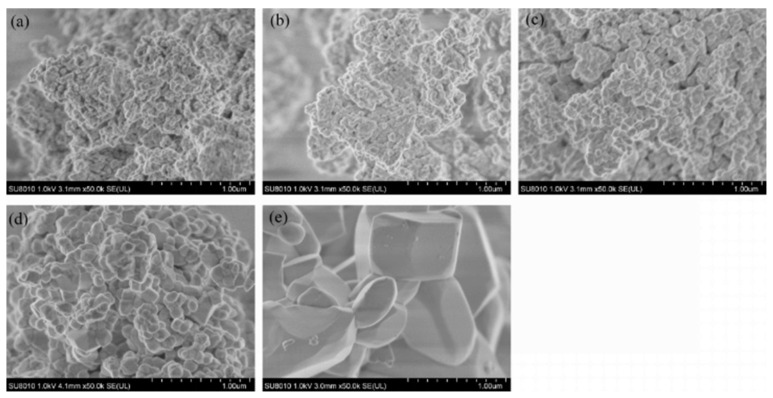
SEM images of YVO_4_: 5%Eu^3+^ at different annealing temperatures: (**a**) 800 °C, (**b**) 900 °C, (**c**) 1000 °C, (**d**) 1100 °C, and (**e**) 1200 °C.

With the increase in annealing temperature, the crystal size gradually increased (Figure 8a–e). When the annealing temperature reached 1100 °C, the crystals transformed rapidly from a thin scaly shape to a spheroid shape, with a uniform size of about 100 nm and excellent dispersion. When annealed at 1200 °C, the crystals exhibited various sizes and shapes, growing rapidly into ingot-like and rod-like forms with micrometer-scale dimensions ranging from approximately 300 nm to 600 nm.

**Figure 9:**
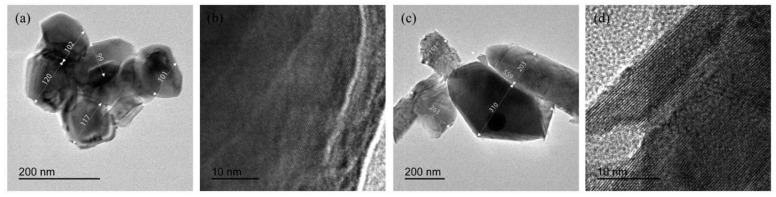
(**a**,**b**) TEM images of YVO_4_: 5%Eu^3+^ annealed at 1100 °C; (**c**,**d**) TEM images of YVO_4_: 5%Eu^3+^, 5%Ba^2+^, 0.5%Bi^3+^ annealed at 1100 °C.

The crystal sizes of YVO_4_: 5%Eu^3+^ were distributed in the range of 100 nm to 120 nm (Figure 9a), while the crystal sizes of YVO_4_: 5%Eu^3+^, 5%Ba^2+^, 0.5%Bi^3+^ were distributed in the range of 150 nm to 310 nm (Figure 9c). All the crystals showed good dispersion. Lattice stripes in the crystals can be clearly observed in Figure 9b,d, indicating that all the crystals possessed good crystallization properties.

### 3.3. Transmittance Spectra

**Figure 10:**
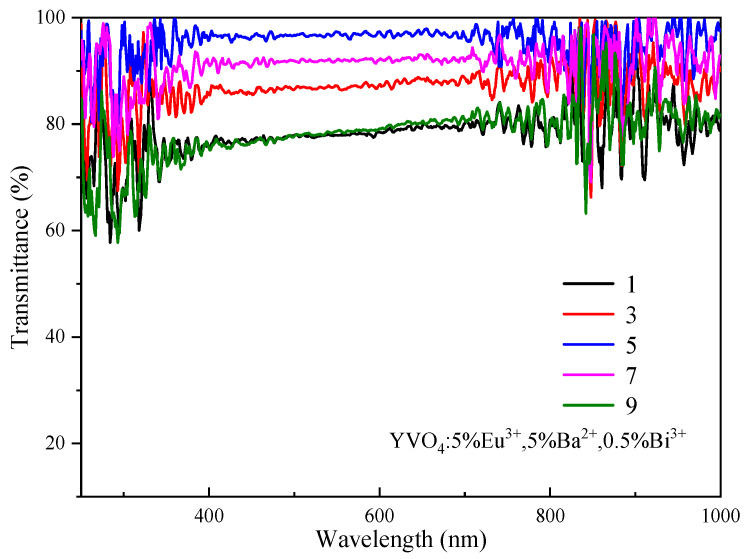
Transmittance spectra of YVO_4_: 5%Eu^3+^, 5%Ba^2+^, 0.5%Bi^3+^ films.

The transmittance of the film initially increased with increase in the number of film layers, and reached the maximum for the three-layer film (Figure 10). Then, the transmittance showed a downward trend as the number of film layers continued to increase. The transmittance of the uniformly coated film fluctuated greatly between 250 and 350 nm. The transmittance in some regions showed an obvious decrease, especially for the small peaks at 260 nm, 280 nm and 320 nm. These reductions were attributed to the absorption of ultraviolet light by the YVO_4_: 5%Eu^3+^, 5%Ba^2+^, 0.5%Bi^3+^ films in this region. For wavelengths between 350 nm and 1000 nm, the transmittance of all films remained above 75%, with the transmittance of three-layer, five-layer and seven-layer films exceeding 85%. The YVO_4_: 5%Eu^3+^, 5%Ba^2+^, 0.5%Bi^3+^ phosphor film developed in this study exhibited high transmittance (>85%) and UV-to-visible light conversion capability in solar cell window layers, demonstrating potential for future exploration in optical temperature sensing applications. Other systems such as Ca_9_Mg_1.5_(PO_4_)_7_: Eu^2+^/Eu^3+^ [50] and Ca_9_Zn_1.5_(PO_4_)_7_: Eu, Tb [51] have also demonstrated the advantages of multi-ion co-doping in temperature sensing and white LEDs. Therefore, the tailored design of multifunctional optical materials can be achieved by modulating doped ion interactions.

## 4. Conclusions

In conclusion, Eu^3+^, Ba^2+^, Bi^3+^ tri-doped YVO_4_ phosphors and thin films were successfully synthesized via a sol–gel approach. The incorporation of Ba^2+^ ions enhanced the luminescence intensity by 2.72 times at 618 nm through lattice expansion and suppression of non-radiative decay, while Bi^3+^ co-doping broadened the excitation bandwidth by 1.39 times via efficient energy transfer. The obtained films exhibit high transparency (>85%) and effective UV-to-red conversion, demonstrating great potential for solar cell applications. This study provides a strategic ternary-doping strategy that simultaneously overcomes the narrow excitation and moderate emission limitations of traditional YVO_4_: Eu^3+^ phosphors. Further studies should focus on thermal stability assessment and device integration to advance practical implementations.

## Data Availability

The original contributions presented in this study are included in the article. Further inquiries can be directed to the corresponding author(s).
